# A patient with an uncommon complication from insertion of a central venous catheter: A case report

**DOI:** 10.1186/1757-1626-1-353

**Published:** 2008-11-26

**Authors:** Imran Khalid, Tabindeh J Khalid, Bruno DiGiovine

**Affiliations:** 1 Division of Pulmonary and Critical Care Medicine, Henry Ford Health System. 2799 W Grand Blvd, Detroit, MI, 48202, USA; 2 Department of Family Medicine, Henry Ford Health System. 1 Ford Place, Detroit, MI, 48202, USA

## Abstract

**Background:**

A 72 year old male was admitted to the medical intensive care unit with septic shock.

**Case presentation:**

A left subclavian central venous catheter was inserted on the day of admission whose tip was pushing against the wall of the vessel lumen. The patient's condition improved with treatment, but three days later had a new episode of acute hypotension. CT scan of the chest showed that the catheter had eroded through the superior vena cava wall.

**Conclusion:**

The catheter was pulled out and patient recovered from the complication with supportive therapy. Care should be taken that the tip of the catheter is in the center of the vessel lumen to avoid this rare, but potentially life threatening, complication.

## Background

Central venous catheters are placed in patients every day in intensive care units, more than 5 million every year just in the United States alone.[1] Infection is the common complication associated with the central venous catheters, and subclavian vein route having the least incidence as compared to femoral or internal jugular routes.[2] The rate of mechanical complications is about 14% which includes failure to place the catheter, arterial puncture, improper position, pneumothorax, hematoma, hemothorax, and asystolic cardiac arrest of unknown etiology.[3] Arrythmias and venous air embolism can also occur.[4,5] We report here an uncommon complication associated with the insertion of central venous catheters.

## Case presentation

**Figure 1:**
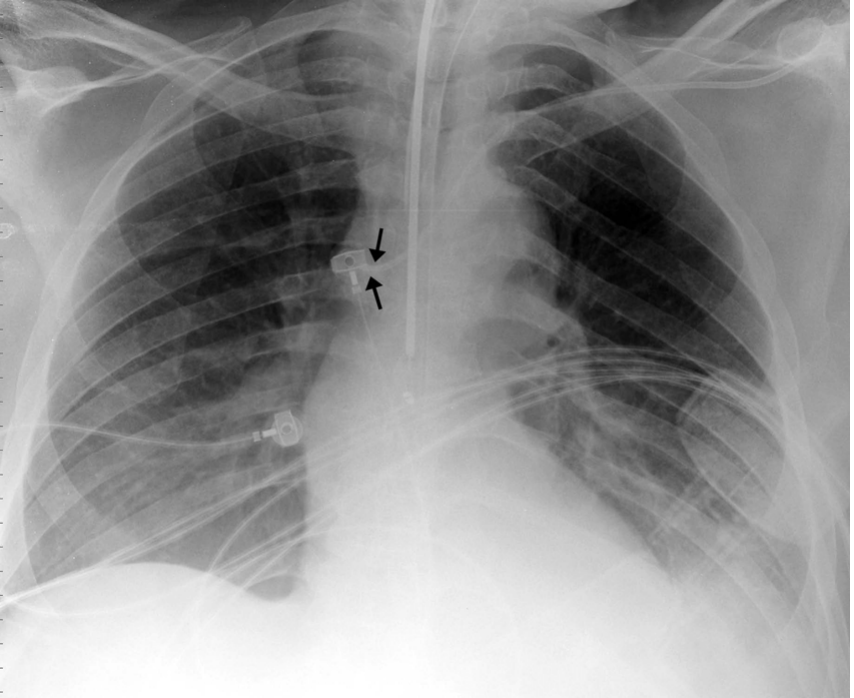
Chest radiograph showing the central venous catheter abutting the superior vena cava.

A 72 year old African American male, with past medical history of hypertension and recently diagnosed diffuse large B-cell lymphoma, was admitted to the hospital for fever and chills that started a day ago. He was discharged from the hospital two days ago on intravenous vancomycin for methicillin resistant Staphylococcus Epidermidis infection. The patient was initially admitted to a general medical floor, but his condition worsened within 24 hours and he went into septic shock and had to be transferred to the medical intensive care unit. He was hypotensive, tachycardic and tachypnic and had evidence of disseminated intravascular coagulation from sepsis. He was endotracheally intubated and placed on mechanical ventilatory support. Blood cultures grew Clostridium Difficile colitis. Appropriate antibiotics were started, and he was treated aggressively with intravenous fluids and also needed norepinephrine infusion for adequate blood pressure maintenance. He had a left subclavian central venous catheter inserted on the day of admission to the intensive care unit for administration of the vasopressor agent (Figure 1: Arrows show the tip of the central venous catheter abutted against the wall of superior vena cava).

**Figure 2:**
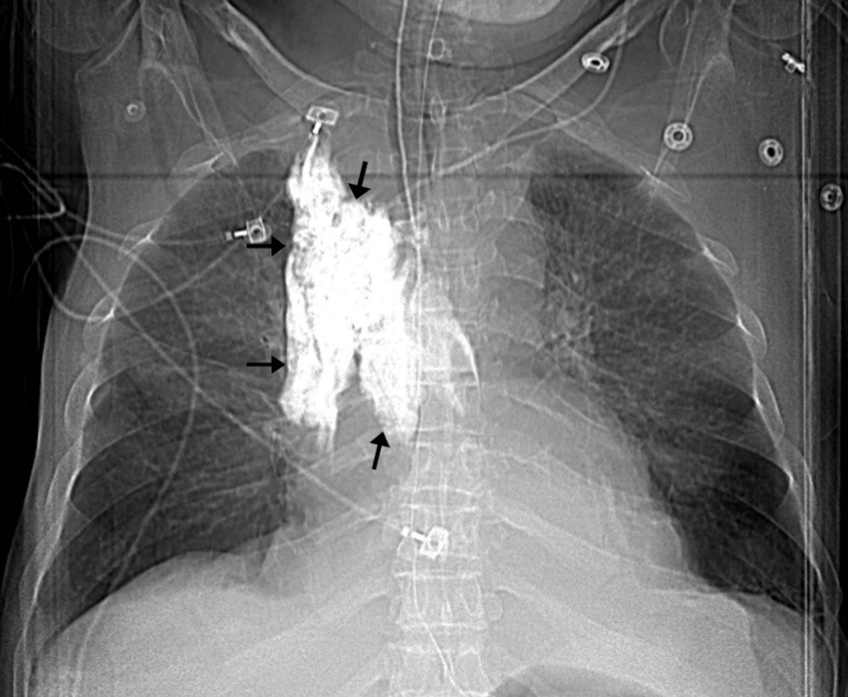
Computed scout image of the chest; arrows showing the extravasated contrast in the superior mediastinum.

**Figure 3:**
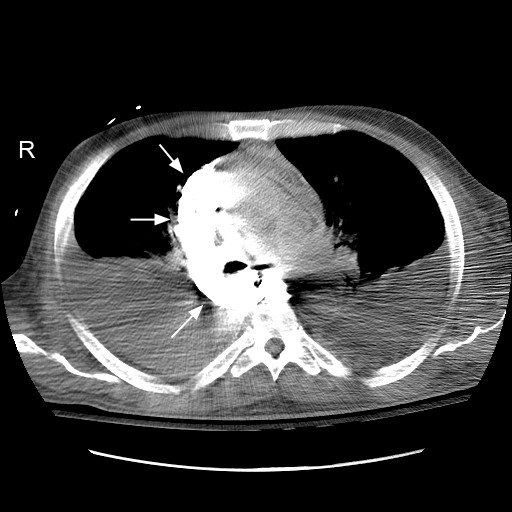
CT scan of chest with arrows showing the contrast in the mediastinum.

The patient's condition improved and he was weaned off the norepinephrine by the third day. On the following day, patient suddenly became hypotensive. While he was being supported by aggressive resuscitative therapy, he had a chest radiograph done as part of the work up to diagnose the etiology of acute deterioration in the clinical condition. The chest radiograph showed widened mediastinum. A subsequent computed tomographic (CT) scan with intravenous contrast given through the left subclavian central venous catheter showed that the catheter had eroded through the wall of superior vena cava and there was extravasated contrast within the superior mediastinum (Figure 2: Computed scout image of the chest; arrows showing the extravasated contrast in the superior mediastinum; Figure 3: CT scan of chest with arrows showing the contrast in the mediastinum). Chest imaging from the day prior did not show these findings. There was no manipulation of the central venous catheter since its insertion. Bilateral chest tubes were inserted which drained bloody fluid. The central venous catheter was pulled out and patient recovered from the complication with supportive therapy. He later died during the same hospitalization from septic shock due to ventilator associated pneumonia from Pseudomonas Aeruginosa.

## Conclusion

The non-traumatic perforation of superior vena cava by central venous catheter tip is relatively rare and has been reported in the catheters inserted via a subclavian approach.[6]

Even though the incidence of the complication is low, nonetheless, care should be taken that the tip of the catheter is in the center of the vessel lumen and is not pushing against the wall to avoid such a complication.

## Competing interests

The authors declare that they have no competing interests.

## Authors' contributions

IK reviewed the patient data regarding the disease and complication and wrote the manuscript. TK did literature search regarding the subject and contributed to a significant portion of the manuscript. BD reviewed the manuscript and made critical revisions. The authors agree with the submission and approve the final manuscript.

## Consent

Written informed consent was obtained from the patient's next of kin for publication of this case report and accompanying images. A copy of the written consent is available for review by the Editor-in-Chief of this journal.
